# Histopathological and ultra structural effects of nanoparticles on rat testis following 90 days (Chronic study) of repeated oral administration

**DOI:** 10.1186/s12951-014-0042-8

**Published:** 2014-10-14

**Authors:** Mansee Thakur, Himanshu Gupta, Dipty Singh, Ipseeta R Mohanty, Ujjwala Maheswari, Geeta Vanage, DS Joshi

**Affiliations:** MGMCET & Departments of Medical Biotechnology, Central Research Laboratory, MGMIHS Sector-1, Kamothe, Navi Mumbai, Maharashtra India; Departments of Medical Genetics, Central Research Laboratory, MGMIHS Sector-1, Kamothe, Navi Mumbai, Maharashtra India; National Center for Preclinical Reproductive and Genetic Toxicology (NIRRH), National Institute for Research in Reproductive Health (ICMR), Jehangir Merwanji Street, Parel, Mumbai, Maharashtra India; Department of Pharmacology, MGM Medical College, MGMIHS, Sector-1, Kamothe, Navi Mumbai, Maharashtra India; Department Pathology, MGM Medical College, MGMIHS, Sector-1, Kamothe, Navi Mumbai, Maharashtra India

**Keywords:** Nanoparticles, Oral exposure, Distribution, Toxicity, Chronic study

## Abstract

**Background:**

Nanoparticles (Ag NPs) have recently received much attention for their possible applications in biotechnology and biomedical. However, little is known about the toxicity in reproductive organs of animal model following exposure to nanoparticles.

**Objective:**

This study therefore, tried to examine the effects of nanoparticles with a diameter range of 5-20 nm on the histology of the testis of wistar rats and correlate it with Transmission Electron Microscopy results.

**Materials and methods:**

Sixteen wistar rats were randomly divided into two groups of 8 rats each. Each group received the following via gavage technique for 90 days: Control Group (Group-1)-tap water; Experimental group (Group 2) - nanoparticles (20ug/kg/day). After ninety days (chronic study), rats were sacrificed and testis tissues was processed for histology and transmission electron microscopic study.

**Results:**

There was significant difference between the observations of group-1 and group 2. The changes observed in the testis were disarray of the spermatogenic cells and disorientation of the testis. These changes were observed to have been disappearing from normal histological features. Detailed structural damages were observed with TEM analysis, such as depletion of germ cells, germinal cells necrosis, especially in spermatogonia and Leydig cells had an abnormal fibroblast-like appearance, abnormal space between neighboring sertoli cells, mitochondria, lost cristae and vacuolated (none energized) with those animals exposed to nanoparticles.

**Conclusion:**

It seems that nanoparticles have acute and significant effects on spermatogenesis and number of spermatogenic cells. More experimental investigations are necessary to elucidate better conclusion regarding the safety of nanoparticles on male reproduction system.

## Introduction

Nanotechnology is a relatively new science; however it already has numerous applications in everyday life, ranging from consumer goods to medicine. Despite the wide application of nanomaterials, there has been a serious lack of information concerning the impact of Nanoparticles on human health and the environment. It is believed that the chemical nature, particle size, morphology, and surface chemistry of nanoparticles are key parameters that influence their toxicity, thus the field of nanotoxicology still lacks the necessary information and clarifications for achieving true risk assessment [1].

Nanoparticles (Ag NPs), is one of the most popular nanomaterial to have been used in material science, such as one of the constituent elements of dental alloys, catheters, implant surfaces and for treating of wounds and burns related infections, as well as in drug delivery in cancer and retinal therapies [2,3]. But, there are some concerns about the safety of using nanoparticles in biomedical and in other industries [4,5]. One concern is about the probable impacts of nanoparticles on remote organs.

Even though it is known that nanoparticles are able to penetrate into reproductive tissue through biological barriers, they may damage various cells: for example, they could reduce sperm numbers, viability and alter cell functions, as well as interfere with embryo development [6]. Although the potential toxic effect of nanoparticles on the reproductive sphere is conceivable, further insights are needed in order to clarify this issue. Theoretically, nanoparticles may have some negative effect on human health and environment and their probable impact(s) on the male reproductive functions is remained to be clarified.

A study by Kim *et al*. reported, that male rats when treated orally with a varying dose of 56 nm nanoparticles for 13 weeks, resulted in decrease in body weight, accumulation of nanoparticles and enlargement of testis(particularly left), and some hepatocyte toxicity was also observed [7].

Further recent confirmation of the potential toxic effects of nanoparticles on spermatogenesis was reported in a study of intravenous administration of nanoparticles in male rats, which observed a size, dose and time-dependent decrease of the epididymal sperm count, increased levels of DNA damage in germ cells and a change in testis seminiferous tubule morphometry [8,9].

Studies on the oral effect of a fixed dose of 1 mg/kg with varying size of nanoparticles on rats for 2 weeks resulted in no significant change in body weight but accumulation in testes was found in rats treated with smaller sized nanoparticles. Study with a varying dose of 42 nm nanoparticles, both alkaline phosphatase and aspartate transaminase serum levels were found to be increased [10].

Finally, the question: 'How do nanoparticles cross the blood testis barrier (BTB)?, plays a vital role in explaining nanoparticles toxicity on spermatogenesis. In comparison with studies involving nanoparticles penetration of the blood brain barrier (BBB), studies involving the BTB are few. Despite the known toxic effects of nanoparticle (SNP), there are a few studies about its influence on reproduction. The motivation of our study was to examine the chronic effects of orally administered single dose of nanoparticles (SNPs) on rat spermatogenesis and seminiferous tubules morphology.

## Materials and methods

### Preparation of nanoparticles

Preparation of 5 ×10^3^ mol/l of nanospheres (puress, Fluka) was done by citrate-reduction route [11] according to the following: 50 ml of 1 mM nitrate was heated to near boiling temperature followed by the addition of 10 ml of 41 mM trisodium citrate solution, stirred vigorously with continuous heating for about 6 minutes. Then, 25 ml of the stock solution was added to 100 ml of double distilled water. The solution was heated until it begins to boil where 5 ml of 1% of sodium citrate was added with vigorous magnetic stirring. Heating was continued till the color of the solution gradually changed to yellow. Then, it was continued for another 15 minutes, after which the solution was removed from the heater and stirred for a further 15 minutes. The electronic UV-Visible absorption spectra were recorded on a Thermo Scientific, Evolution 201 series spectrophotometer, using a quartz cell with a path length of 1 cm.

Transmission electron microscopy [Tecnai 12BT FE1 120 kV TEM] at a voltage of 120 kV was used to study the particles size, and morphology of the nanoparticles where the aqueous dispersion of the nanoparticles was drop cast onto a carbon coated copper grid, and the grid was air dried at room temperature before viewing under the microscope, and the diameter was determined from the micrographs.

### Animal and treatment

Male wistar rats (150–200 gms & 10 -12 weeks old) of the wistar strain were obtained from the Animal House, Haffkin Institute. They were maintained on a standard pellet diet and tap water and were kept in polycarbonate cages with woodchip bedding under a 12:12 h light: dark cycle and room temperature 22–24°C. Rats were acclimatized to the environment for 2 weeks prior to experimental use. This study was conducted following the guidelines of the Animal Ethics Committee, MGM Institute of Health Sciences. Rats were randomly divided into 2 groups (n =08). The animals were assigned to each experimental group, with 8 animals per group. Animals in each group were marked with picric acid on their head, abdomen, tail, back, left upper leg and left hind leg for easy identification and were subjected to the following treatment:

- Group 1 - Control group of animals were administered double distilled water orally using oral gavage technique once a day for 90 days.
- Group 2 –Experimental group of animals were administered nanoparticles (20/kg of 5-20 nm particle size) orally using the oral gavage technique once a day for 90 days.

### Histopathological studies

These studies were carried out at the Pathology laboratory of MGM College, Kamothe. At the end of the feeding schedule (90 days) the animals were sacrificed by an overdose of chloroform. The tissue of interest, testis, were immediately fixed in 10% buffered neutral formalin solution. The tissue embedded in molten paraffin with the help of metallic blocks, covered with flexible plastic moulds and kept under freezing plates to allow the paraffin to solidify. Cross sections (5 μm thick) of the fixed tissue were cut using microtome (Leica PM 2125, Germany). These sections were then stained with hematoxylin and eosin method (Gurr [12]) and visualized under light microscope (Magnus, India) to study the microscopic architecture of the testis.

### Transmission electron microscopic analysis

Testis tissues was cut into small 1 mm^3^ pieces, and immediately fixed in modified Karnovsky’s fixative (4% glutaraldehyde +4% paraformaldehyde +0.2% picric Acid +0.02% calcium chloride +0.2 M cacodylate buffer), dehydrated in graded acetone solutions, and embedded in resin (araldite). Rinsing, post fixation, dehydration and infiltration was carried out in the KOS microwave tissue processor. Semi-thin sections were cut at 1 μm in thickness with a glass knife using Leica Ultracut R Ultramicrotome. They were stained with 1% toluidine blue dissolved in 1% borax for approximately 30 m at 40–50°C, washed, dried and examined under the light microscope for general orientation. Ultra thin sections, below 100 nm in thickness were obtained from the selected areas using Leica Ultracut R Ultramicrotome (Leica Microsystems, Milton Keynes, England). The grids were then stained with 2% alcoholic uranyl acetate for 10 min in the dark, thoroughly washed in milliq water and allowed to air dry before examination. The sections were then viewed and photographed using the FEI TECNAI™ Transmission Electron microscope (120kv) TEM was carried out at *National Institute For Research In Reproductive Health* (*NIRRH*), an ICMR institute, Parel.

## Results

### Synthesis of nanoparticles

**Figure 1:**
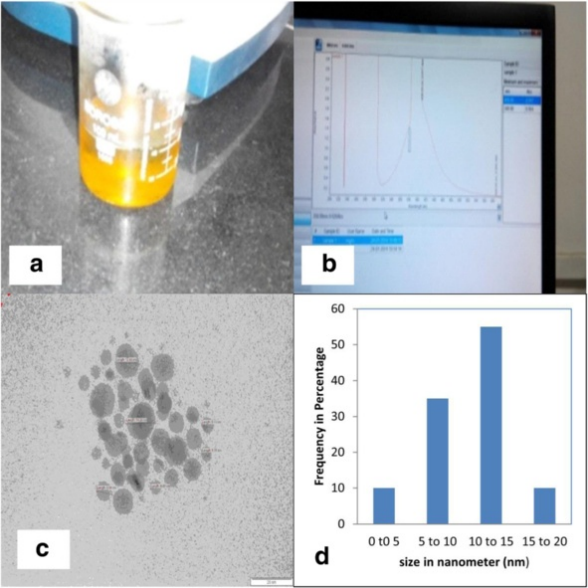
**Synthesis and Characterization of Silver Nanoparticles. (a)** Bright yellow colour of synthesized nanoparticles (λmax – 397 nm), **(b)** UV–Vis absorption spectrum of nanoparticles obtained, **(c)** TEM analysis of Ag < 25 nanoparticles (120KV) representative micrograph showing nanoparticles and **(d)** Histogram representing the number-based distribution of the mean core diameter of the nanoparticles. Scalebar:20 nm.

Silver nanoparticles were synthesized according to the method described in the previous section, the solution turned light yellow after the addition of 2 ml of silver nitrate and a brighter yellow (Figure 1a) when all of the silver nitrate had been added and indicating that the silver nanoparticles were formed. UV-visible spectroscopy is one of the most widely used techniques for structural characterization of nanoparticles. The absorption spectrum (Figure 1b) of the pale yellow colloids prepared by borohydrate reduction showed a Surface Plasmon Absorption band with a maximum of 397 nm indicating the presence of spherical or roughly spherical nanoparticles, and TEM imaging confirmed this (Figure 1c). This image show agglomerates of small grains and some dispersed nanoparticles. The particle size histograms of particles (right-hand illustration in Figure 1d) show that the particles range in size from 5 to 20 nm.

### Body and organ weights

No deaths were observed and no other signs of toxicity were apparent in male rats treated orally with nanoparticles (20 μg /kg body weight) for 90 days. All rats were weighed weekly and also testis from nanoparticles exposed rats showed no significant differences in weight in comparison to the control groups (data not shown). Furthermore, no behavioral differences were seen.

### Histopathological results

#### Control group/group 1

**Figure 2:**
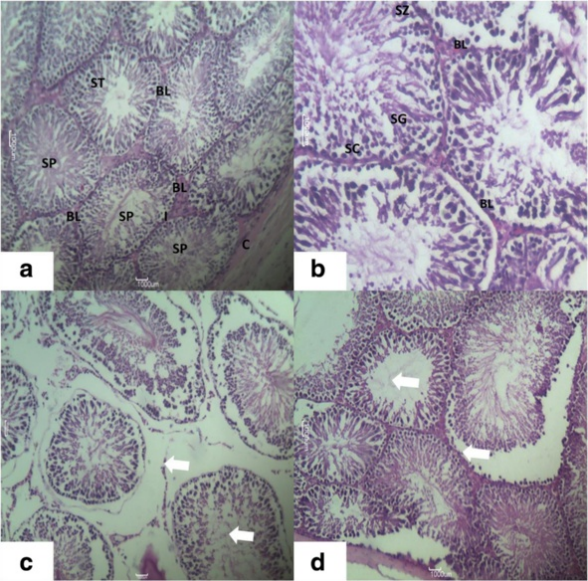
**Photomicrograph of transverse section of H&E staining of rats. (a)** Testis of the Control Group Showing: the Capsule (C) Enclosing Normal Seminiferous Tubules (ST) appearing Circular or nearly circular, their Basal Lamina (BL) with Clumps of Interstitial Leydig Cells (I). Spermatozoa (SP), seen within the Tubular Lumen. (H & E; X400), **(b)** Testis of the Control Group Showing: Part A Seminiferous Tubule Resting on Basal Lamina (BL) and Shows Spermatogonia (SG), Spermatozoa (SZ) and Spermatid. Sertoli Cells (SC) are Seen Lying Perpendicular to the Basement Membrane (H & are E; X660) and **(c,d)** Testis of experimental group of rat showing disorganization of the normal appearance of the testis with atrophy in the seminiferous tubule, disorganization of germinal epithelium, and loss of spermatogenic cells. 400X.

Sections of the testis stained by H & E in control groups (Figure 2a, b) showed a thick fibrous capsule (tunica albuginea) enclosing a number of adjacent Seminiferous Tubules (ST) separated by interstitial cells. The seminiferous tubules appeared as rounded or oval surrounded by a thin Basal Lamina (BL). The tubules were lined by stratified germinal epithelium, which consists of two distinct populations of cells; the spermatogenic cells and the sertoli cells. Sertoli cells appeared as elongated cells, with irregular poorly defined outline and oval basal nuclei. The spermatogenic cells represented the different stages of spermatogenesis, with the spermatogonia resting on the basal lamina and having small and dark nuclei. Spermatocytes appeared as large cells with large oval nuclei. Inner to primary spermatocytes, there were the secondary spermatocytes with their relatively smaller size followed by spermatids and Spermatozoa (SP). The spermatids were detected at their different steps of spermatogenesis. The cells first appeared rounded with central rounded nuclei (round spermatids) and gradually, they become elongating spermatids that form the spermatozoa with their characteristic shape. In-between the tubules and the interstitial tissue are present blood vessels with clusters of cells with ovoid or polygonal shape and spherical nuclei representing the leydig cell.

#### Experimental group (nanoparticles Treated/Group 2)

Group 2 rats fed with 20 /kg/day for 90 days showed following observations:- H & E stained sections of the testis in group 2 treated with nanoparticles (Figure 2c) showed histological changes that were at variance with those obtained in the control. There was disorganization of the normal appearance of the testis with overall different degrees of atrophy in the seminiferous tubules. At the same time a disorganization of the germinal epithelium, with loss of the spermatogenic cells specially spermatocytes and spermatids and exfoliation of the germ cells was also observed. In the seminiferous tubular lumen and almost all tubules showed severe atrophy as they were devoid of epithelium, with only sertoli cells and spermatogonia present within the depleted tubules. Sperms were hardly seen. The spermatogenic cells showed degeneration and/or necrosis. Testis treated with nanoparticles confirmed the atrophic changes found in the capsule and wall of the seminiferous tubules which was observed in H & E stained sections.

### Electron microscopic observation

#### Basal lamina and spermatogonia

**Figure 3:**
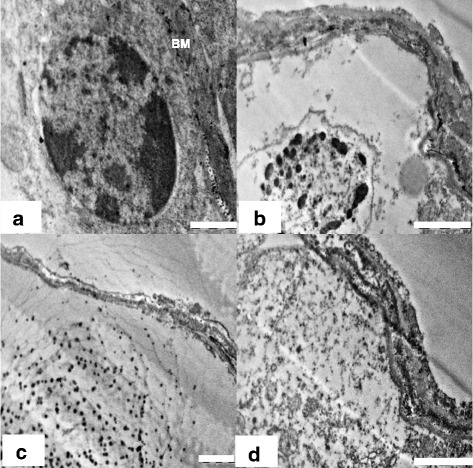
**Electron micrograph images (TEM) of basement membrane and spermatogonia of rat. (a) Group** 1(control) showing normal basement membrane (BM), and spermatogonia (SG). Scale bar =1 μm, **(b)** Group 2 showing signs of inflammatory damage of testicular tissue in the form of irregular and thickened basal lamina and apoptotic spermatogonial cells. Scale bar =2 μm, **(c)** Group 2 showing accumulation of nanoparticles near basement membrane (*). Scale bar =2 μm and **(d)** Group 2 showing degenerated cytoplasm of seminiferous epithelium. Scale bar =2 μm.

Ultra structural observation of the control rats (Group 1) showed normal testicular architecture with thick basal lamina, surrounded by myoid cells and regularly arranged spermatogonia. The spermatogonial cell had a spherical or oval nucleus (Figure 3), finely granular nucleoplasm, scanty cytoplasm, and granular ribonucleic protein particles. They were in close contact with basal lamina with nuclei lying parallel to tubular limiting membrane. In nanoparticles treated group (Group 2), the seminiferous tubules were more thickened with fibrous connective tissue than in control groups (Figure 3b). It showed strongly damaged irregular tubular basement membrane with finger like extensions towards the cytoplasm. The group 2 demonstrated cellular alteration and apoptosis in germ cells (Figure 3b). It was evident that spermatogonia cells were separated from basal lamina and neighboring cells by remarkable spaces. The nuclear envelope showed dissruption in spermatogomium. There was evident accumulation of Nanoparticles near the basement membrane (Figure 3c). The clumped chromatins, degenerated cytoplasm and nucleus in spermatogonial cells were seen in the treated animals (Figure 3b, d)

#### Sertoli cell cytoplasm and nucleus

**Figure 4:**
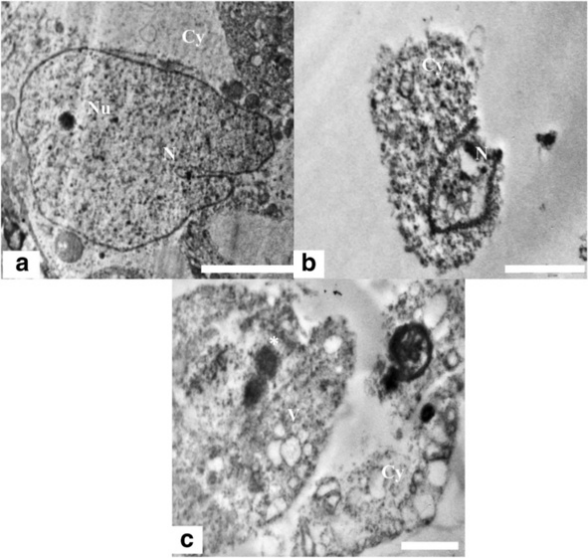
**TEM micrograph of Sertoli cell nucleus and Cytoplasm. (a)** Testis ultra structure of the control animals showing the sertoli cell nucleus (N) and cytoplasm (Cy). The nucleus shows indentation and a prominent nucleolus (Nu). Scale bar =2 μm, **(b)** The animals in experimental group 2 showing apoptotic Sertoli cell nucleus. Scale bar =2 μm and **(c)** Ultra structure of highly vacuolated (V) and degenerated sertoli cell having electron dense bodies (*).Scale bar =2 μm.

**Figure 5:**
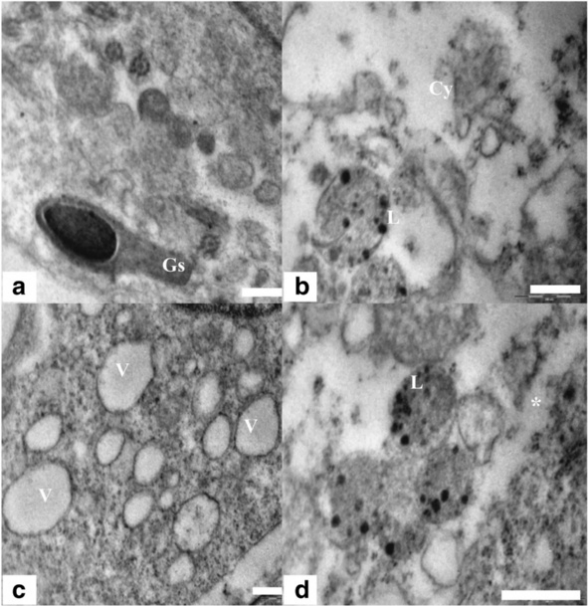
**TEM micrograph of Sertoli cell, lysosome and Cytoplasm. (a)** Sertoli cell cytoplasm of control group 1 showing healthy cytoplasm having intact germ cells (Gs) and intact granular cytoplasm (Cy). Scale bar =0.5 μm, **(b)** Sertoli cell ultra structure showing degenerated cytoplasm (Cy) and lysosome bound nanoparticles (L). Scale bar =0.5 μm, **(c)** Highly vacuolated (V) sertoli cell cytoplasm in nanoparticle treated group. Scale bar =0.5 μm and **(d)** Sertoli cell cytoplasm in treated group 2 showing accumulation (*) of nanoparticles and liposomes containing nanoparticles (L). Scale bar =0.5 μm.

The ultra structure of the control animals showed healthy sertoli cells with oval shaped large nucleus, prominent nucleolus and indentation (Figure 4a). Sertoli cells cytoplasm was extended from the basal lamina to the lumen of the seminiferous tubules and enveloped the adjacent germinal elements (Figure 5a). Prosecretory granules, rosettes of glycogen granules and free ribosome were scattered throughout the cytoplasm. It was evident that the sertoli cells of the Group 1 were normal, with clear nuclear membrane and no evident morphological abnormalities in cellular organelles. In contrast, Group 2 showed alterations in sertoli cells nucleus and cytoplasm. The sertoli cell nuclei were found necrotic and dislocated from the basal portion (Figure 4b). The cytoplasm was degenerated with several vacuoles and electron dense materials (Figure 4c). There were degenerative changes in form of necrosis and severe vacuolization were seen in sertoli cell cytoplasm at the ultra structural level in nanoparticles treated group 2 (Figure 5b, c). Abundance of nanoparticles containing lysosomal bodies was noticed in sertoli cell cytoplasm (Figure 5b, d). Accumulation of nanoparticles was also observed in the cytoplasm (Figure 5d).

#### Spermatocytes and round spermatids

**Figure 6:**
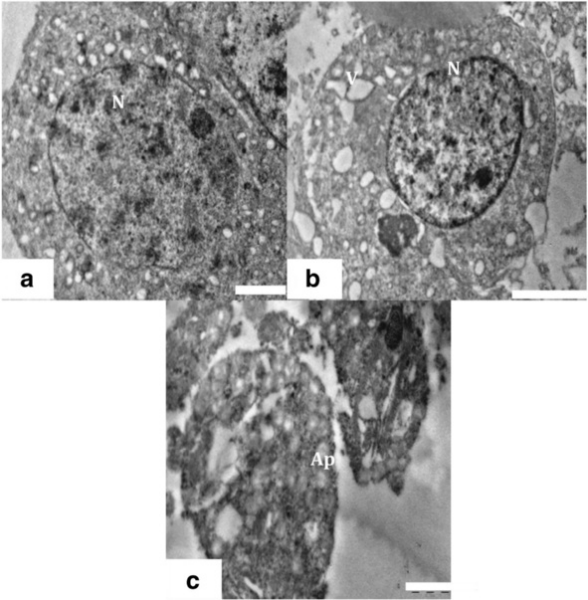
**TEM of Spermatocyte. (a)** Ultra structure of spermatocyte from control group 1 with prominent well defined nucleus (N) granular cytoplasm. Scale bar =2 μm, **(b)** Spermatocyte showing degenerated and highly vacuolated cytoplasm in experimental group. Scale bar =2 μm and **(c)** Ultra structure of apoptotic spermatocyte (Ap) from experimental group. Scale bar =1 μm.

**Figure 7:**
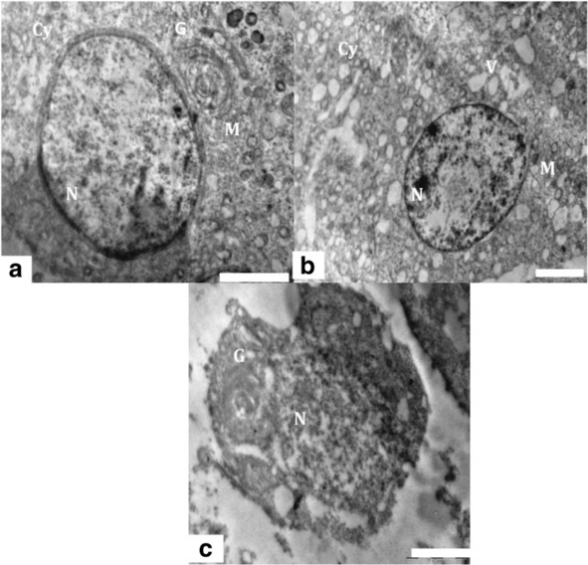
**TEM of Spermatid. (a)** Ultra structure of the testis of control animals, showing a round spermatid having distinct nucleus (N), mitochondria (M) and golgi vesicle (G). Scale bar =2 μm, **(b)** Testis of experimental animal showing degenerated cytoplasm (Cy), vacuoles (V) and collapsed mitochondria (M). Scale bar =2 μm and **(c)** Ultra structure of apoptotic round spermatid showing collapsed golgi vesicles (G) and nucleus (N) from experimental group. Scale bar =2 μm.

A large number of spermatocytes and round spermatids were noticed in the luminal part of seminiferous tubules in control group 1 animals. The spermatocytes displayed round configurations with prominent nuclei (Figure 6a). The nuclei had distinct chromatin networks and well-defined nuclear membranes. The cytoplasm of the spermatocytes appeared granular, characterized by dispersed mitochondria and loose networks of rough endoplasmic reticulum. Round spermatids had characteristic well-defined nuclei with distinct nuclear membranes and chromatin networks, and their cytoplasm was occupied majorly with mitochondria (Figure 7a). Formation of acrosome marked by presence of golgi vesicles, appeared normal (Figure 7a). The cytoplasmic organelles of spermatocytes and spermatids of the control group 1 had no evident morphological abnormalities. The ultra structural alterations were observed in testes of rats treated with nanoparticles (Figures 6b and 7b). During the late stages of apoptosis, the structure of the nuclear envelope was dispersed, accompanied by chromatin condensation and marginalization in spermatocytes (Figure 6c). The apoptotic germ cells were identified with condensed nuclear chromatin and degenerated cytoplasmic organelles. The mitochondria underwent collapse of cristae (Figure 7b) and the structure of the nuclear envelope disappeared (Figure 7c) in round spermatids. Experimental group 2 germ cell showed many vacuoles and residual particles in their cytoplasm resulting from organelle degeneration (Figures 6c and 7c).

#### Elongating and elongated spermatid

**Figure 8:**
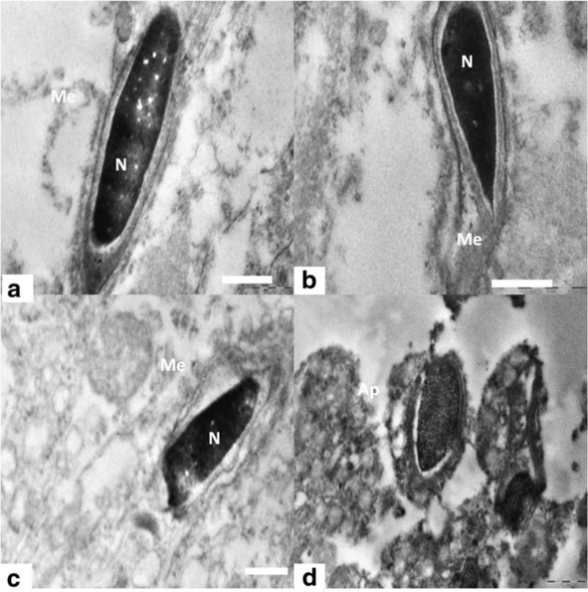
**TEM of Elongating spermatids. (a)** Electron micrograph showing elongating spermatids of control group 1 showing intact acrosome, cell membrane (Me) and condensed nucleus (N). Scale bar =0.5 μm, **(b)** Elongating spermatids from nanoparticle treated group 2 showing perturbed membrane (Me) and acrosome. Scale bar =0.2 μm, **(c)** Detachment of the inner acrosomal membrane (Me) from the nuclear envelope. Scale bar =1 μm and **(d)** Apoptotic spermatid showing collapsed and vacuolated membrane around the nuclear periphery. Scale bar =1 μm.

**Figure 9:**
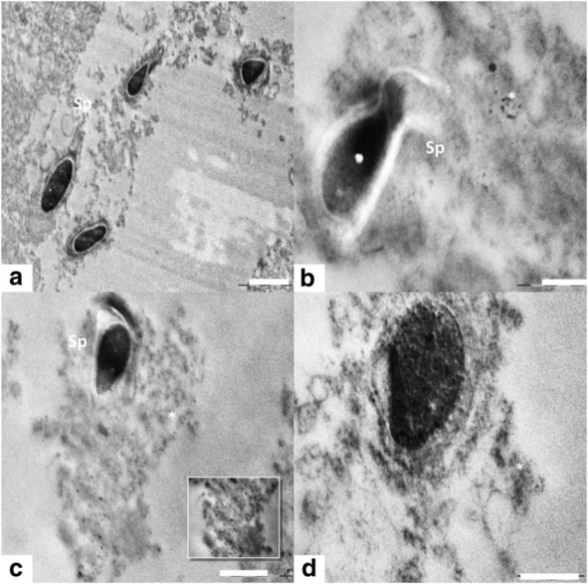
**TEM of spermatogenesis. (a)** Normal spermatogenesis showing healthy round and elongating spermatids (Sp) in control group. Scale bar =2 μm, **(b)** Abnormally developed spermatid (Sp) engulfed by sertoli cell cytoplasm and nanoparticle accumulation (*) in experimental group. Scale bar =0.5 & 1 μm, **(c)** Abnormally developed spermatid (Sp) engulfed by sertoli cell cytoplasm and nanoparticle accumulation (*) in experimental group. Scale bar =0.5 & 1 μm and **(d)** Deposition of nanoparticle on acrosomal membrane and sertoli cell cytoplasm. Scale bar =0.5 μm.

**Figure 10:**
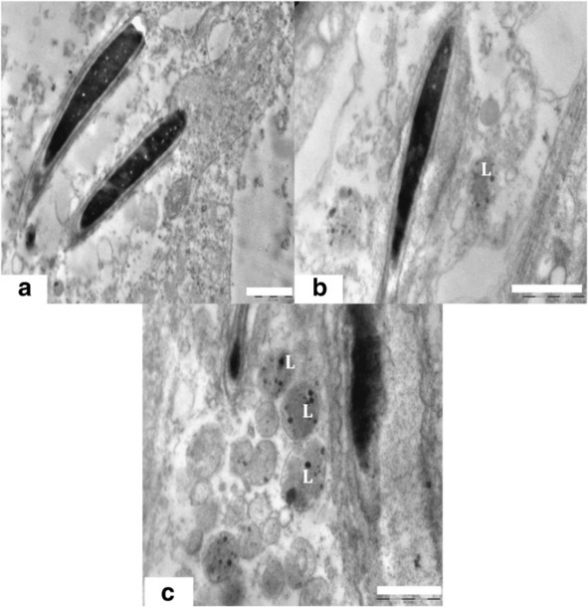
**TEM of spermatids & lysosomal bodies. (a)** Ultra structure of a normal spermatid with intact head, acrosomes and membrane. Scale bar =1 μm, **(b)** Presence of lysosomal bodies containing nanoparticles near the elongating spermatids and deformed acrosomal membrane in experimental group. Scale bar =1 μm and **(c)** Sertoli cell cytoplasm crowded with lysosomal bodies containing nanoparticles and abnormal spermatids. Scale bar =1 μm.

**Figure 11:**
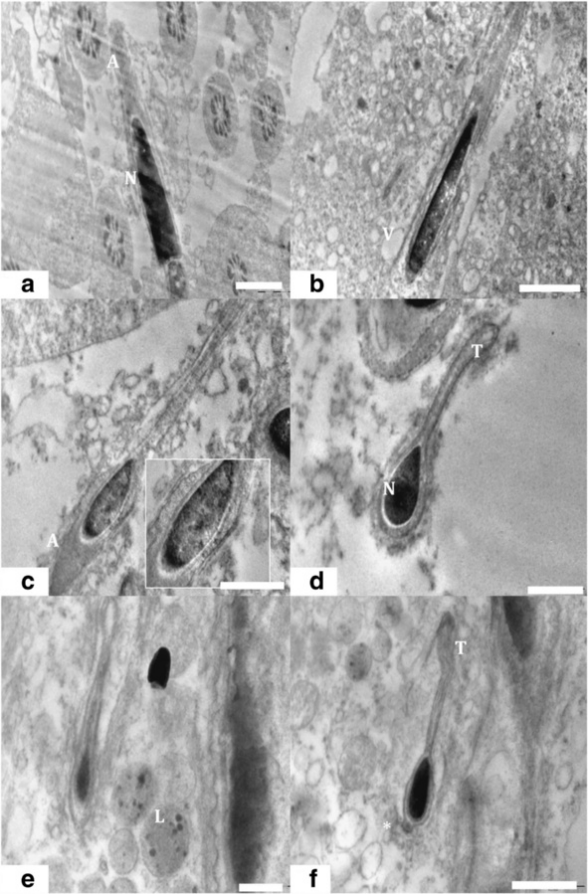
**TEM of elongated spermatid. (a)** Ultra structure of the testis of rat from control group 1 showed intact acrosome (A), nucleus (N) and other cellular architecture of elongated spermatid. Scale bar =1 μm, **(b)** Experimental group 2 treated with Nano particle showed severely damaged ultra structure of elongated spermatid with high vacuolation (V) and electron dense bodies. Scale bar =2 μm, **(c)** Elongated spermatid from treated group 2 having membrane abnormalities surrounded by degenerated sertoli cell cytoplasm. Scale bar =1 μm, **(d)** Elongated spermatid from control group 1 with intact head and tail (T). Scale bar =0.5 μm, **(e)** Elongated spermatid from experimental group 2 deformed tail and head with increased sub acrosomal space. nanoparticle entrapped in lysosmal bodies (L) near the elongated spermatid was also noted. Scale bar =0.5 μm and **(f)** Elongated spermatid containing deformed acrosomal membrane (*) and tail (T) were commonly observed. Scale bar =1 μm.

Electron micrograph of control group 1 showed different stages of elongating and elongated spermatids with intact ultra structure. Control group 1 spermatozoa presented normal ultra structure cells with the typical shape of head, intact cell membranes, acrosomes, and homogenous nuclei (Figures 8a, 9a, 10a and 11a, d). Intact plasma lemma, inner and outer acrosomal membranes, electron dense homogenous contents of acrosomes was also noted. By contrary the experimental group 2 showed spermatids having several ultra structural deformities. The large population of the developing spermatids showed deleterious abnormalities. Among the major abnormalities noted were acrosomal changes, sub acrosomal swelling, perturbed integrity of the membranes (Figure 8b, c). Many of the apoptotic spermatids were noticed which had vacuolated and collapsed membrane around the nucleus (Figure 8d). Electron micrographs showed that deformed elongating spermatids were phagocytized by sertoli cell, which resulted in engulfed spermatids and residual bodies in the cytoplasm of sertoli cells (Figure 9b, c). nanoparticles were also found accumulated in the sertoli cells cytoplasm (Figure 8b, c). Deposition of nanoparticles could also clearly observe on the acrosomal membrane of elongating spermatids (Figure 9d). There were evident crowding of lysosomal bodies containing nanoparticles and large number of abnormal spermatids in sertoli cell cytoplasm of experimental group. The lysosomal bodies containing nanoparticles were found present very near to the elongating spermatids and deformed acrosomal membrane in experimental group 2 were also evident (Figure 10b, c). Experimental group 2 showed heavily damaged spermatid with high vacuolation and electron dense bodies in the sertoli cell cytoplasm (Figure 11b). The sertoli cell cytoplasm surrounding the elongated spermatid was found degenerated (Figure 11c). Ultra structure of elongated spermatid from experimental group 2 had deformed tail and head with increased sub acrosomal space. Lysosmal bodies containing nanoparticle were also noticed near the elongated spermatid (Figure 11e). Deformed acrosomal membrane and tail were very commonly seen in elongated spermatid (Figure 11f).

## Discussion

Several particle features, such as type, size, zeta potential, dispersion/agglomeration status, as well as potential interaction with biomolecules, influence nanoparticles toxicity and hence their effects in humans. Generally, size (and hence the surface/mass ratio) has been considered as the most important factor for the toxicity of nanoparticles [13]. In the present study, we attempted to use a nanosize particle to study the toxicity. Chithrani and Chan [14] reported that small particle size does not necessarily lead to better uptake and hence increased toxicity [14]. Additionally, it has been reported that the surface chemistry of nanoparticles influence interparticle interactions, hence particle distribution and in effect transport across membranes [15] and genotoxicity [16].

To the best of our knowledge, this work is the first to investigate the effect of bio distribution of nanoparticles in testicular cells of wistar rat having exposure for 90 days. No abnormal effects like physical, body weight and behavioural were noted in both control and treated male rats. Previously, the liver and kidneys have been described as the primary organs for distribution, whether the exposure was orally [7,17-19], intravenously [20,21], subcutaneously [22] or through inhalation [23]. Two other oral exposure studies reported high distribution of to the testis for 28 days exposure and sub-chronic [17,19,24].

Data from the present study suggest that continuous exposure of male rats to nanoparticles for 90 days causes histopathological changes such as epithelial cell sloughing, atrophic changes and decrease in germ cell numbers due to cytotoxicity were the factors seen in treated animals. Degenerative changes in the seminiferous tubules and decrease of spermatozoa in the testis, epididymis and vas deferens are the evidence for genotoxicity. Degenerative changes in the seminiferous tubules indicate that nanoparticles may directly interfere in the process of spermatogenesis.

In 2011, Tiwari and colleagues used 4 doses of nanoparticles 4, 10, 20 and 40 mg/kg which were injected intravenously and they concluded that nanoparticles in doses less than 10 mg/kg are safe for biomedical application and has no side effects, but doses more than 20 mg/kg are toxic [25]. This study is contradictory to our findings but cannot be held against as the size range of the nano particles used is not mentioned and dose used in the study was different than that used in our present study. It may be emphasized that even a low dose of SNP (20 ug/kg) [26,27], as used in the present study, is capable of produced ultra structural changes. However, It would have been interesting if these ultrastructrual changes could be correlated to any functional changes if any produced by these nanoparticles. However, in absence of such data, it may be speculated that it is the size rather than dose that has a major impact on the degree of tissue damage.

However, another study mentioned that after 2 weeks of intravenous administration of nanoparticles in mice, no obvious acute toxicity were observed with the dose of 30 mg/kg, while inflammatory reactions in lung and liver cells were induced in mice treated with the dose of 120 mg/kg [28]. It should be added, that besides the doses and size of particles, the duration of exposure and route of administrationof nanoparticles may be responsible for the large variation in the toxicity profile of nanoparicles observed in various studies [29]. The oral route should cause less toxicity as compared to the parenteral routes of administration as the bioavailability of nanoparticles would be the least via this route. The oral route exposes the nanoparticles to thigh first pass metabolism resulting in low systemic levels that may produce toxicity subsequently.

Spermatogenesis is a highly orchestrated and dynamic process resulting in the continual production of spermatozoa in mammals. Our transmission electron microscopic results indicated that severe cellular changes occurred in the cytoplasm of spermatogenia, primary and secondary spermatocytes, round and elongating spermatids and Sertoli cells in the experimental group, which indicates the alterations in the function of these cells [30]. Sertoli cells from the blood-testis barrier which is important in maintaining sperm formation and the micro-environment around the germ cells. They also involved in production of of growth factors and nutrients [31], which serve as energy sources and aid the maturation of sperm that enter into the lumen. In our study, we found huge accumulation of nanoparticles in the sertoli cell cytoplasm which might have induce toxicity in these cells which would have affected the nutritional intake and normal maturation of spermatogenic cells at various stages. Nanoparticles also induced degenerative changes in basement membrane which maintains the structural and functional integrity of testicular tissues. Altered basement membrane structure might lead to severe functional impairment of the testis [32]. The ultra structural observations suggested that all animals exposed to nanoparticles had apoptosis in different germinal cells such as spermatogonia, spermatocytes and spermatids leading to depletion of germ cells. The germ cells apoptosis that occurred in the testicular epithelium served as a major cause to reduce the germ cell population in the nanoparticles treated animals. Nanoparticles injured or disrupted the functions of basement membrane, sertoli cells and spermatogonial cells resulting in an increase in the elimination of the germ cell numbers via apoptosis [31]. Observation of detached germ cells, deformed spermatocytes and spermatids, may be due to the marked disruption of the sertoli-germ cells interaction. The disruption in this physical interaction might have led to the sloughing of the germ cells from seminiferous epithelium [33].

## Conclusion

The present Study clearly indicates that nanoparticles (20 ug/kg) have deleterious effects on testicular structure of rats. Ultra structural observations presented the evidence of severely impaired and apoptotic germ cells in the testis. Therefore, nanoparticles cannot be considered as a therapeutic hope drugs that require exposure for longer duration. Further studies are required for dose lower than the one used in the present study (20 ug/kg) for strengthening this observation. From the results of this study, we concluded that nanoparticles led to a deleterious effect on the testis that increased progressively with time. The results obtained from this study provided important clues about the impact of nanoparticles on reproductive health and would be of great value in assessing its health hazards.
